# Elucidating Drug-Like Compounds and Potential Mechanisms of Corn Silk (*Stigma Maydis*) against Obesity: A Network Pharmacology Study

**DOI:** 10.3390/cimb43030133

**Published:** 2021-11-06

**Authors:** Ki-Kwang Oh, Md. Adnan, Dong-Ha Cho

**Affiliations:** Department of Bio-Health Convergence, College of Biomedical Science, Kangwon National University, Chuncheon 24341, Korea; nivirna07@kangwon.ac.kr (K.-K.O.); mdadnan1991.pharma@gmail.com (M.A.)

**Keywords:** corn silk (*Stigma Maydis*), obesity, network pharmacology, PPAR signaling pathway, PI3K-Akt signaling pathway

## Abstract

Corn silk (*Stigma Maydis*) has been utilized as an important herb against obesity by Chinese, Korean, and Native Americans, but its phytochemicals and mechanisms(s) against obesity have not been deciphered completely. This study aimed to identify promising bioactive constituents and mechanism of action(s) of corn silk (CS) against obesity via network pharmacology. The compounds from CS were identified using Gas Chromatography Mass Spectrometry (GC-MS) and were confirmed ultimately by Lipinski’s rule via SwissADME. The relationships of the compound-targets or obesity-related targets were confirmed by public bioinformatics. The signaling pathways related to obesity, protein-protein interaction (PPI), and signaling pathways-targets-bioactives (STB) were constructed, visualized, and analyzed by RPackage. Lastly, Molecular Docking Test (MDT) was performed to validate affinity between ligand(s) and protein(s) on key signaling pathway(s). We identified a total of 36 compounds from CS via GC-MS, all accepted by Lipinski’s rule. The number of 36 compounds linked to 154 targets, 85 among 154 targets related directly to obesity-targets (3028 targets). Of the final 85 targets, we showed that the PPI network (79 edges, 357 edges), 12 signaling pathways on a bubble chart, and STB network (67 edges, 239 edges) are considered as therapeutic components. The MDT confirmed that two key activators (β-Amyrone, β-Stigmasterol) bound most stably to PPARA, PPARD, PPARG, FABP3, FABP4, and NR1H3 on the PPAR signaling pathway, also, three key inhibitors (Neotocopherol, Xanthosine, and β-Amyrone) bound most tightly to AKT1, IL6, FGF2, and PHLPP1 on the PI3K-Akt signaling pathway. Overall, we provided promising key signaling pathways, targets, and bioactives of CS against obesity, suggesting crucial pharmacological evidence for further clinical testing.

## 1. Introduction

Obesity is a serious health issue worldwide because it is involved in the main causes of comorbidity and mortality, including diabetes, hypertension, heart failure, atherosclerosis, and some cancers [1,2]. Obesity is characterized by the accumulation of excessive adipose tissues in the body, leading to energy imbalance, alteration of appetite hormones, and insulin resistance [3,4]. Clinically, the criteria of obesity is the that Body Mass Index (BMI) is equal to 30.0 or higher [5]. Obesity can present at all ages, globally, a report announced that the number of overweight and obese individuals will be projected to be 1.35 billion and 573 million by 2030 [6,7].

**Figure 1:**
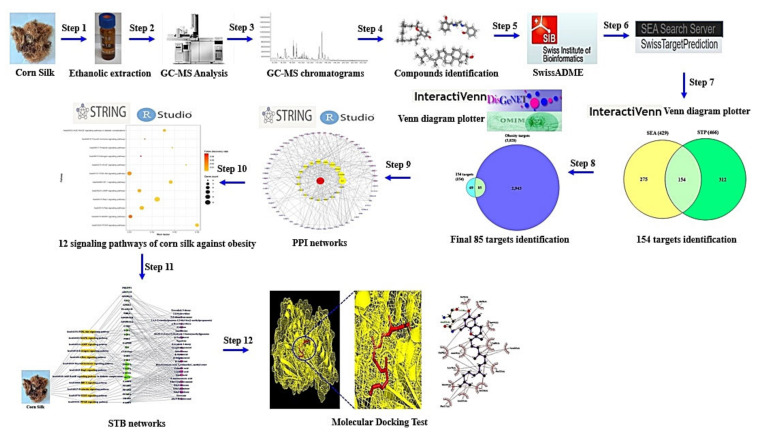
Research process of network pharmacology analysis of CS against obesity.

The most optimal therapeutic strategy against obesity is to inhibit the accumulation of fat in the body as well as to suppress the appetite with special medication [8,9]. At present, a representative drug of anti-obesity is Orlistat (PubChem ID: 3034010), used to decrease the absorption of fatty acid in intestine by inhibiting gastric and pancreatic lipase [10]. In addition, some medications (diethylpropion, fenfluramine, sibutramine, rimonabant) with appetite suppression efficacy have been prescribed to alleviate obesity in most countries [11]. However, most anti-obesity drugs have serious adverse events such as steatorrhea, flatulence, headache, and hypoglycemia [12]. Natural herbal plants are good resources with less side effects, compared to synthetic drugs [13]. Most recently, osmotin is characterized by a natural plant protein with antifungal efficacy, which is homologous functionally to adiponectin for preventing an excess of fatty acids in the body [14,15]. However, even though these are derived from herbal plants, protein drugs are susceptible to degradation and are not given orally due to poor bioavailability [16]. Some anti-obesity natural organic small compounds (<500g/mol) have been isolated from marine sponges: Palinurin (from *Ircinia variabilis*) [17], Dysidine (from *Dysidea villosa*) [18], Questinol and citreorosein (from *Stylissa flabelliformis*) [19], and Phorbaketal A (from *Phorbas* sp.) [20]. Other resources are land herbal plants with diverse anti-obesity organic small compounds: Curcumin (from *Curcuma longa* rhizome), Carnosic acid and carnosol (from *Salvia officinalis* leaves), Epigallocatechin 3-O gallate (from *Camellia sinensis*), Ursolic acid (from *Actinidia arguta* root), and Crocetin and crocin (from *Gardenia jasminoides* fruits) [21]. Currently, the majority of drug candidates in herbal plants are dependent on their main parts such as leaves, roots, and fruits. On the other hand, we suggest that medicinal utilization of agricultural substances is a good approach to identify their value. Of these, a report demonstrated that some flavonoids and phenolics from the 50% ethanolic corn silk (CS) extracts have potent anti-obesity efficacy, leading to anti-adipogenesis and lipolysis [22]. However, commonly, bioavailability improvement of phenolic compounds including flavonoids should be applied to accomplish pharmacological functions through leading-edge delivery system [23]. From this point of view, we need to establish a new methodology and concept to analyze anti-obesity on CS. At present, drug-like compound(s), target(s), and signaling pathway(s) of CS against obesity have not been reported. Thus, the studies on drug-like compounds and promising mechanism(s) of CS against obesity should be strengthened to provide pharmacological evidence to support its therapeutic application in alleviating obesity. Network pharmacology is a significant methodology to elucidate multiple components such as signaling pathways, targets, and compounds [24]. Network pharmacology is a key to decipher multiple targets of herbal bioactive compounds [25]. With the rapid progression of network pharmacology, the unveiling of interaction between multi-components and multi-targets gives us a clue to illustrate pathogenesis [26]. Moreover, the network pharmacology analysis in holistic perspectives is an effective approach to develop compounds for the treatment of metabolic disorders such as diabetes mellitus (DM), and obesity [25]. The aim of this study is to investigate the signaling pathways, targets, and compounds of CS against obesity. Firstly, compounds from ethanolic CS extract have been identified by Gas Chromatography-Mass Spectrometry (GC-MS) and screened by Lipinski’s rule to identify Drug Like Compounds (DLCs). Then, targets related to DLCs or obesity collected using public bioinformatics, and overlapping targets between DLCs and obesity targets were identified. Secondly, the protein-protein interaction (PPI) based on overlapping targets was constructed by RPackage. Next, a bubble chart used to visualize the Rich factor on overlapping targets was built by RPackage. Thirdly, relationships between signaling pathways, targets, and DLCs were visualized by RPackage. Finally, Molecular Docking Test (MDT) was performed to understand the best affinity between targets and DLCs on key signaling pathways. The concise workflow is exhibited in Figure 1.

## 2. Materials and Methods

### 2.1. Plant Material and Extracts Preparation

Corn silk (CS) were collected from (latitude: 36.683084, longitude: 128.512617), Gyeongsangbuk-do, Korea, in July 2021. The CS were dried in a shady zone at room temperature (20–22 °C) for 7 days, and dried CS powder was made using an electric blender. Approximately 20 g of CS powder was soaked in 1000 mL of 100% ethyl alcohol (Daejung, Siheung city, Gyeonggi-do, Korea) for 15 days and repeated 3 times to achieve a high yield rate. The solvent extract was collected, filtered with Whatman filter paper No. 1 (Whatman, Model no. WF1-1850, UK Maidstone) and evaporated using a vacuum evaporator (IKA- RV8, Staufen city, Germany) at 40 °C. The yield after evaporating was 1.98 g (Yield rate: 0.99%), which was calculated as follows:

Yield (%) = (Dried CS weight/Evaporated extraction weight) × 100

### 2.2. GC-MS Analysis Condition

Agilent 7890A (Agilent, Santa Clara, CA, USA) was used to perform GC-MS analysis. GC was equipped with a DB-5 (30 m × 0.25 mm × 0.25 µm) capillary column (Agilent, Santa Clara, CA, USA). Initially, the instrument was maintained at a temperature of 100 °C for 2.1 min. The temperature rose to 300 °C at a rate of 25 °C/min and was maintained for 20 min. Injection port temperature and helium flow rate were ensured as 250 °C and 1.5 mL/min, respectively. The ionization voltage was 70 eV. The samples were injected in split mode at 10:1. The MS scan range was set at 35–900 (*m*/*z*). The fragmentation patterns of mass spectra were compared with those stored in the W8N05ST Library MS database (analyzed 7 September 2021). The percentage of each compound was calculated from the relative peak area of each compound in the chromatogram [27].

### 2.3. GC-MS Compounds in CS and Screening of DLCs

The chemical constituents in CS were detected via GC-MS analysis, which were input into PubChem (https://pubchem.ncbi.nlm.nih.gov/, accessed on 9 September 2021) to identify SMILES (Simplified Molecular Input Line Entry System) format. The screening of DLCs is based on Lipinski’s rule via SwissADME (http://www.swissadme.ch/) (accessed on 9 September 2021). Additionally, topological polar surface area (TPSA) to measure cell permeability of compounds was identified by SwissADME (http://www.swissadme.ch/, accessed on 9 September 2021). Commonly, its cut-off value to evaluate cell permeability is typically less than 140 Å^2^ [28].

### 2.4. Identification of Target Proteins Associated with Bioactives or Obesity

The bioactives confirmed by Lipinski’s rule put the SMILE format into two two public cheminformatics: Similarity Ensemble Approach (SEA) (accessed on 10 September 2021) [29] and SwissTargetPrediction (STP) (accessed on 10 September 2021) [30] with “*Homo Sapiens*” mode. The relationship between target proteins and bioactives were obtained by the two cheminformatics, which demonstrated their use as significant tools to be validated experimentally: A total of 80% out of the novel drug candidates line up with the SEA result, and the promising target proteins of cudraflavone C were identified through STP, thereby, its biological activities were validated by the experiment [31,32]. Altogether, we confirmed that novel potential ligands and target proteins would be identified using the validated data. The target proteins related to obesity were collected by two public bioinformatics DisGeNET (https://www.disgenet.org/search, accessed on 13 September 2021) and OMIM (https://www.ncbi.nlm.nih.gov/omim) (accessed 13 September 2021). The overlapping target proteins between DLCs from CS and obesity-related target proteins were identified and visualized on InteractiVenn [33]. Then, we visualized it on Venn Diagram Plotter.

### 2.5. PPI Construction of Final Target Proteins and Identification of Rich Factor

The interaction of the final overlapping target proteins was identified by STRING analysis (https://string-db.org/, accessed 14 September 2021) [34]. The number of nodes and edges were identified by PPI construction and thus, signaling pathways involved in overlapping target proteins were explicated by the RPackage bubble chart illustration. On the bubble chart, two key signaling pathways of CS against obesity were finalized.

### 2.6. The Construction of STB Network

The STB networks were visualized as a size map, based on a degree of value. In the network map, green rectangles (nodes) represented the signaling pathways; yellow triangles (nodes) represented the target proteins; red circles (nodes) represented the bioactives. The size of the yellow triangles stood for the number of relationships with signaling pathways; the size of red circles stood for the number of relationships with target proteins. The assembled network was constructed by utilizing RPackage.

### 2.7. Bioactives and Target Proteins Preparation for MDT

The bioactives related to the two key signaling pathways were converted. sdf from PubChem into. pdb format utilizing Pymol, and thus they were converted into. pdbqt format via Autodock. The number of the six proteins on the PPAR signaling pathway, i.e., PPARA (PDB ID: 3SP6), PPARD (PDB ID: 5U3Q), PPARG (PDB ID: 3E00), FABP3 (PDB ID: 5HZ9), FABP4 (PDB ID: 3P6D), and NR1H3 (PDB ID: 2ACL), and the number of the seven proteins on PI3K-Akt signaling pathway, i.e., AKT1 (PDB ID: 3O96), IL6 (PDB ID: 4NI9), VEGFA (PDB ID: 3V2A), PRKCA (PDB ID: 3IW4), FGF1 (PDB ID: 3OJ2), FGF2 (PDB ID: 1IIL), and PHLPP1 (not available in the PDB) were identified on STRING via RCSB PDB (https://www.rcsb.org/, accessed 16 September 2021). The proteins were chosen as. PDB format were converted into. pdbqt through Autodock (http://autodock.scripps.edu, accessed on 17 September 2021).

### 2.8. MDT of Bioactives on Target Proteins Related to Two Key Signaling Pathways

The ligand molecules were docked with target proteins using autodock4 by setting-up 4 energy range and 8 exhaustiveness as default to obtain 10 different poses of ligand molecules [35]. The center of each target protein on PPAR signaling pathway was PPARA (x = 8.006, y = −0.459, z = 23.392)), PPARD (x = 39.265, y = −18.736, z = 119.392), PPARG (x = 2.075, y = 31.910, z = 18.503), FABP3 (x = −1.215, y = 46.730, z = −15.099), FABP4 (x = 7.693, y = 9.921, z = 14.698).

The center of each target protein on PI3K-Akt signaling pathway was Akt1 (x = 6.313, y = −7.926, z = 17.198), IL6 (x = 11.213, y = 33.474, z = 11.162), VEGFA (x = 38.009, y = −10.962, z = 12.171), PRKCA (x = −14.059, y = 38.224, z = 32.319), FGF1 (x = 9.051, y = 22.527, z = −0.061), FGF2 (x = 26.785, y = 14.360, z = −1.182), PHLPP1 (x = −3.881, y = 1.398, z = 2.661). The active site’s grid box size was x = 40 Å, y = 40 Å, z = 40 Å. The detailed information of 2D binding was identified by LigPlot^+^ 2.2 (https://www.ebi.ac.uk/thornton-srv/software/LigPlus/, accessed 18 September 2021) [36]. After MDT, bioactives with the lowest Gibbs free energy were selected to depict the bioactive-protein complex in Pymol.

## 3. Results

### 3.1. Physicochemical Properties of Chemical Compounds from Corn Silk (CS)

**Figure 2:**
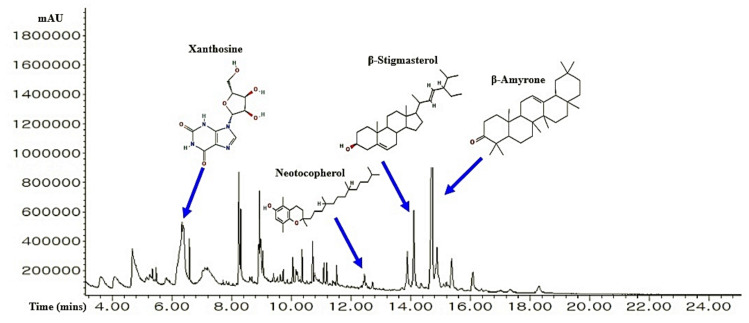
A typical GC-MS peaks of CS ethanolic extract and the number of four key bioactives.

A total of 36 chemical compounds from CS were detected through GC-MS analysis (Figure 2), and compound name, retention time, peak area, PubChem ID, and taxonomic classification are presented in Table 1. All 36 chemical compounds were accepted by Lipinski’s rule (Molecular Weight ≤ 500 g/mol; Moriguchi octanol-water partition coefficient ≤ 4.15; Number of Nitrogen or Oxygen ≤ 10; Number of NH or OH ≤ 5), including TPSA value (< 140 Å^2^) (Table 2).

### 3.2. Identification of Overlapping Target Proteins between SEA and STP Linked to 36 Compounds

**Figure 3:**
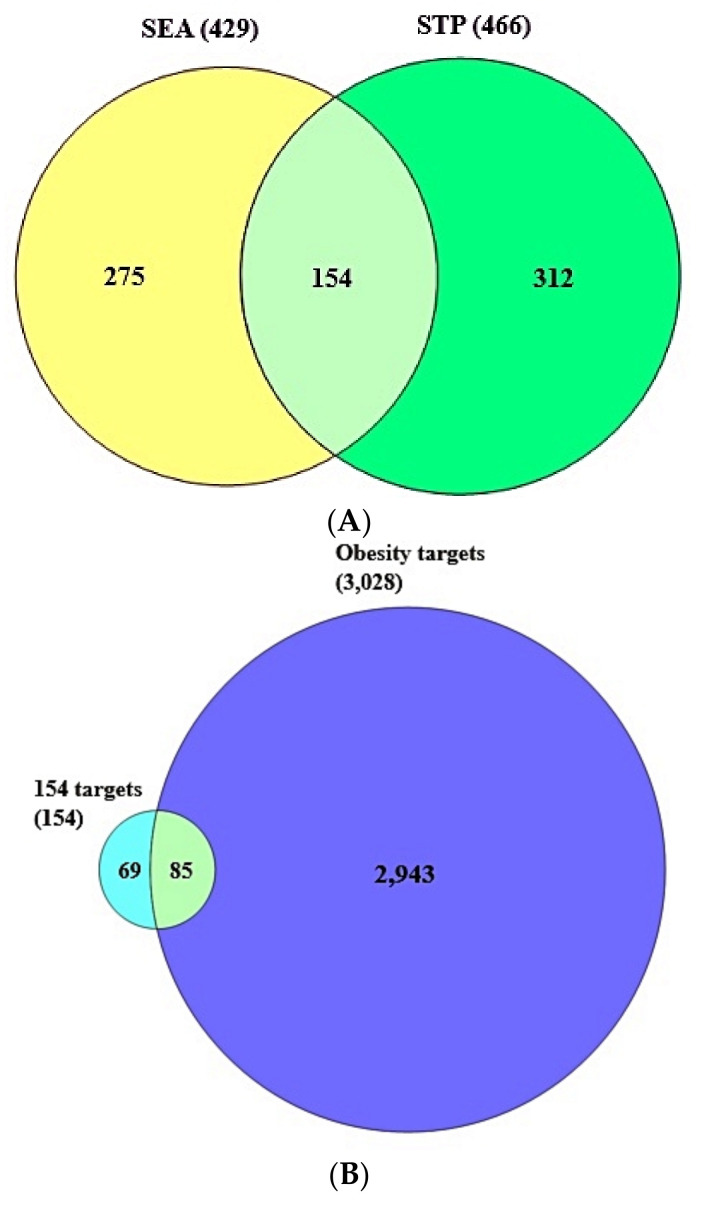
(**A**) A total of 154 overlapping targets between SEA (429 targets) and STP (466 targets). (**B**) A total of 85 final targets between the 154 overlapping targets and obesity-related targets (3028 targets).

A total of 429 target proteins from SEA and 466 target proteins from STP linked to the abovementioned 36 compounds were identified through SMILES format (Supplementary Table S1). The results of the Venn diagram exhibited that 154 overlapping target proteins were overlapped between SEA and STP public databases (Supplementary Table S1) (Figure 3A).

### 3.3. The Final Overlapping Target Proteins between Obesity-Related Target Proteins and the 154 Overlapping Target Proteins

As shown in Supplementary Table S2, a total of 3028 target proteins associated with obesity were retrieved by DisGeNet and OMIM databases. The Venn diagram displayed that a total of 85 target proteins overlapped between obesity related to 3028 target proteins and 154 overlapping target proteins (Supplementary Table S2) (Figure 3B).

### 3.4. Protein-Protein Interaction (PPI) from Final 85 Target Proteins

**Figure 4:**
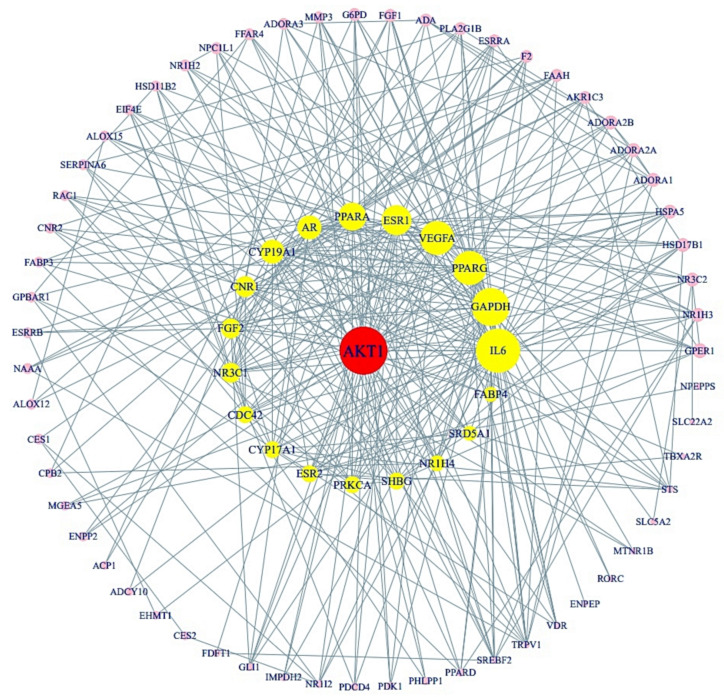
PPI networks (79 nodes, 357 edges). The size of the circle represents degree of values.

Using STRING analysis, 79 out of 85 target proteins were correlated closely with each other with 79 nodes and 357 edges (Figure 4). The eliminated 6 target proteins (RNASE2, SLC22A6, GSTK1, PAM, OXER1, and THRA) did not interact with the 85 target proteins. In the PPI network, the AKT1 target protein had the greatest degree of centrality (43) and was considered as the hub target protein (Table 3).

### 3.5. The 12 Signaling Pathways and Identification of Two Key Pathways of CS against Obesity

**Figure 5:**
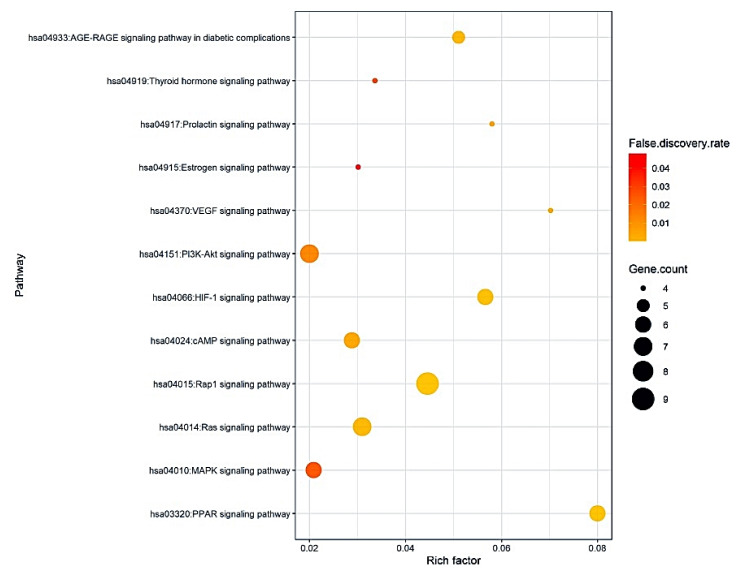
A bubble chart of 12 signaling pathways associated with progression and development of obesity.

The results of Kyoto Encyclopedia of Genes and Genomes (KEGG) pathway enrichment analysis showed that 85 target proteins were related directly to 12 signaling pathways (False Discovery Rate < 0.05). The 12 signaling pathways were implicated with occurrence and development of obesity, suggesting that these pathways might be important signaling pathways of CS against obesity. The description of the 12 signaling pathways was represented in Table 4. In addition, a bubble chat suggested that both the PPAR signaling pathway with the highest rich factor and PI3K-Akt signaling pathway with the lowest rich factor might be key signaling pathways of CS against obesity (Figure 5).

### 3.6. The Construction of a Signaling Pathway-Target Protein-Bioactive (STB) Networks

**Figure 6:**
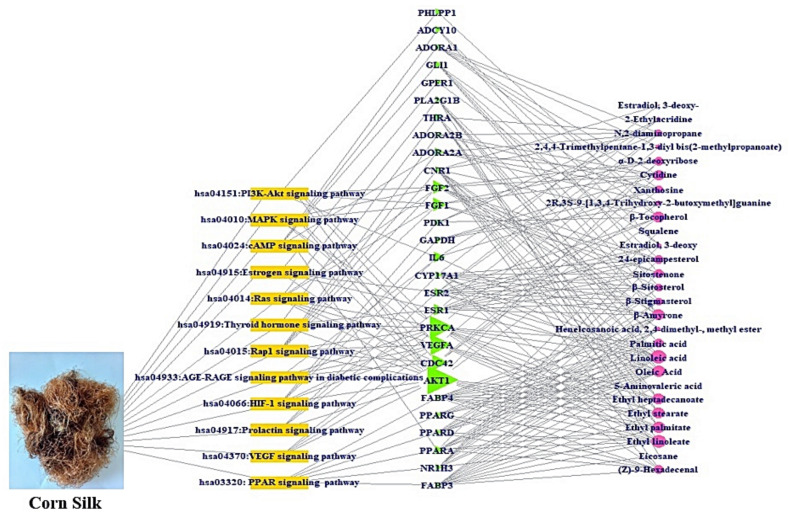
STB networks (67 nodes, 239 edges). Yellow rectangle: signaling pathway; green triangle: target; pink circle: bioactive.

A signaling pathway-target protein- bioactive (STB) network of CS was exhibited in Figure 6. There were 12 signaling pathways, 28 targets, and 27 bioactives (67 nodes, 239 edges). The nodes stood for a total number of each component: signaling pathways, target proteins, and bioactives. The edges represent relationships of the three components. The STB network indicated that each component of the network is a significant element with therapeutic efficacy against obesity. The AKT1 is the uppermost target with the greatest degree value (11) among 12 signaling pathways (Table 5). Noticeably, a sole signaling pathway not to be connected to AKT1 was the PPAR signaling pathway with the highest rich factor.

### 3.7. MDT of 6 Target Proteins, 2 Key Bioactives, and 9 Positive Controls on PPAR Signaling Pathway

Through MDT analysis, it was unveiled that PPARA(PDB ID: 3SP6) was associated with 9 bioactives: (1)β-Amyrone, (2) Squalene, (3) Ethyl palmitate, (4) Heneicosanoic, 2,4-dimethyl-,methyl ester, (5) Oleic acid, (6) Ethyl linoleate, (7) Palmitic acid, (8) Linoleic acid, and (9) (Z)-9-Hexadecenal, PPARD (PDB ID: 5U3Q) was related to 8 bioactives: (1) β-Stigmasterol, (2) β-Sitosterol, (3) Heneicosanoic, 2,4-dimethyl-,methyl ester, (4) Ethyl linoleate, (5) Linoleic acid, (6) Oleic acid, (7) Palmitic acid, and (8) (Z)-9-Hexadecenal, PPARG (PDB ID: 3E00) was connected to 6 bioactives: (1) β-Amyrone, (2) Ethyl linoleate, (3) Linoleic acid, (4) Oleic acid, (5) Palmitic acid, and (6) (Z)-9-Hexadecenal, FABP3 (PDB ID: 5HZ9) was associated with 12 bioactives: (1) β-Amyrone, (2) Heneicosanoic, 2,4-dimethyl-,methyl ester, (3) Eicosane, (4) Ethyl stearate, (5) Ethyl heptacecanoate, (6) Ethyl linoleate, (7) Ethyl palmitate, (8) Linoleic acid, (9) (Z)-9-Hexadecenal, (10) Oleic acid, (11) Palmitic acid, and (12) 5-Aminovaleric acid, FABP4 (PDB ID: 3P6D) was related to 11 bioactives: (1) β-Amyrone, (2) Heneicosanoic, 2,4-dimethyl-,methyl ester, (3) Ethyl stearate, (4) Ethyl palmitate, (5) Ethyl heptacecanoate, (6) Ethyl linoleate, (7) 5-Aminovaleric acid, (8) Oleic acid, (9) Linoleic acid, (10) Palmitic acid, and (11) (Z)-9-Hexadecenal, and NR1H3 (PDB ID: 2ACL) was connected to 9 bioactives: (1) β-Amyrone, (2) β-Stigmasterol, (3) β-Sitosterol, (4) Estradiol, 3-deoxy, (5) Sitostenone, (6) 24-epicampesterol, (7) Ethyl linoleate, (8) Linoleic acid, and (9) Oleic acid.

**Figure 7:**
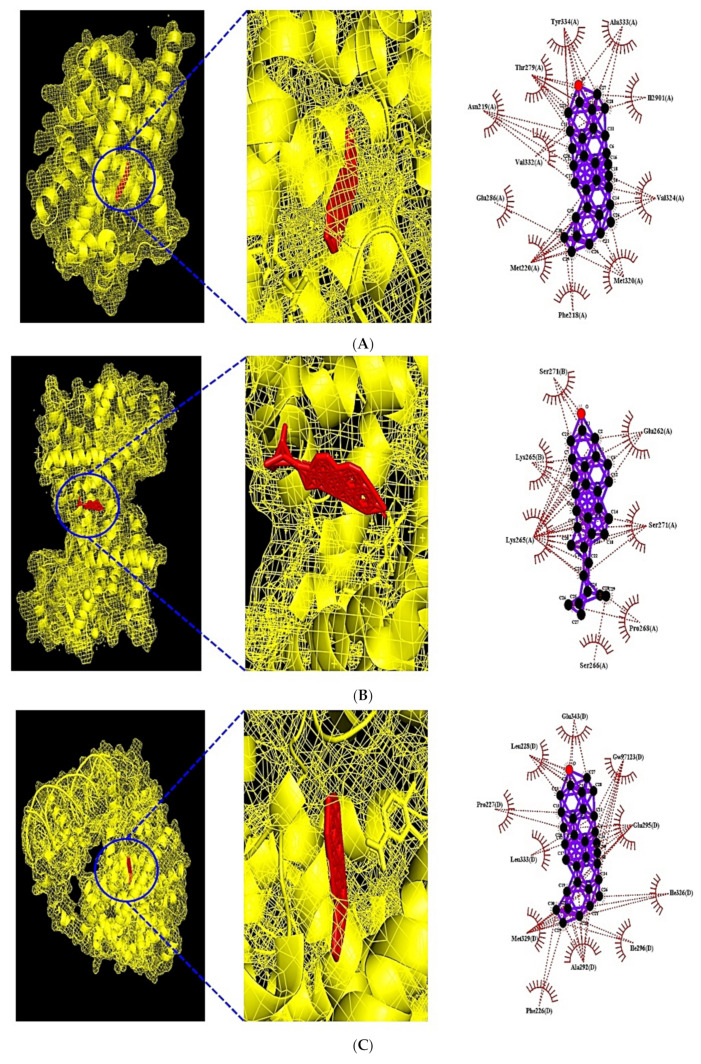

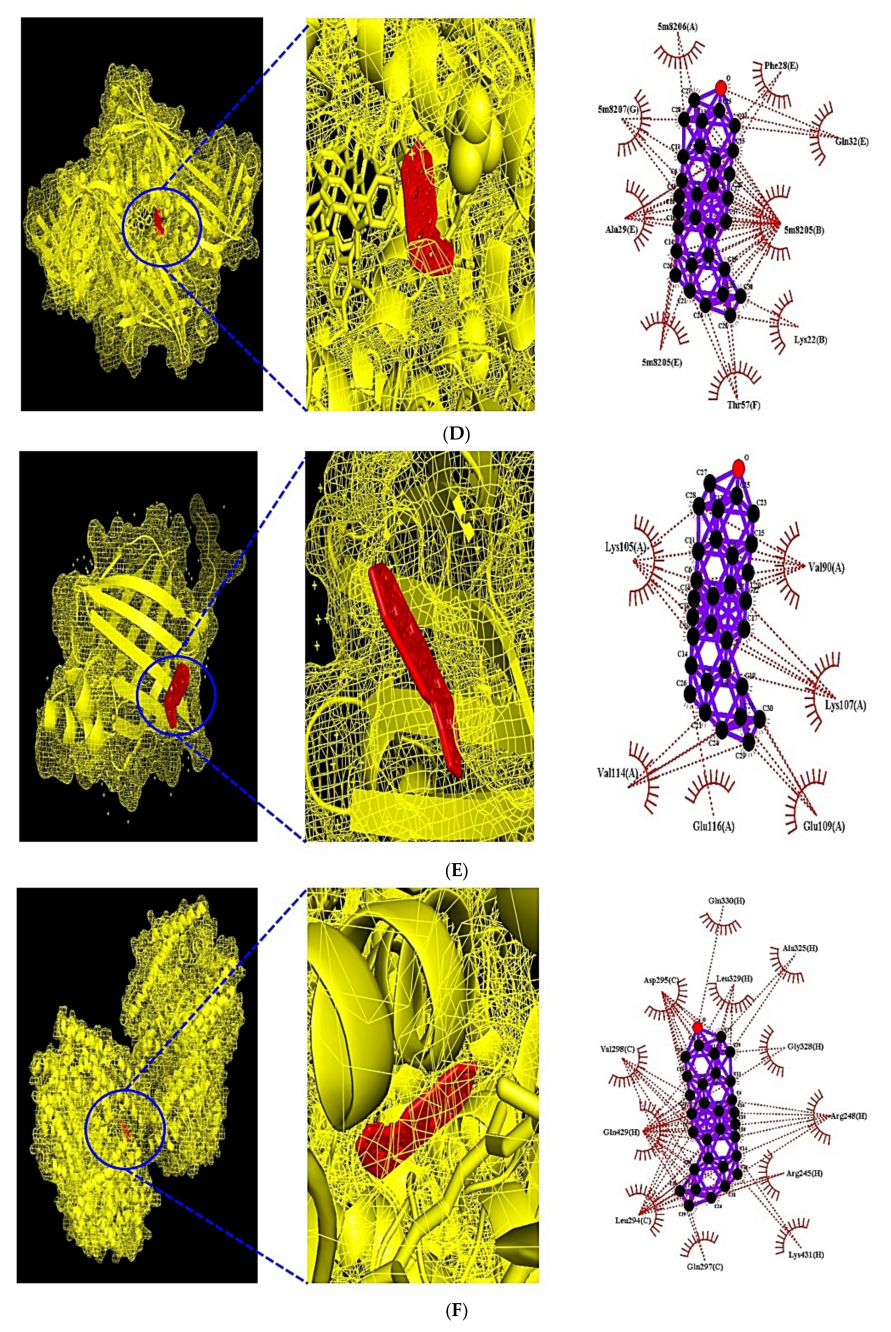
(**A**) MDT of β-Amyrone (PubChem ID: 612782) on PPARA (PDB ID: 3SP6). (**B**) MDT of β-Stigmasterol (PubChem ID: 6432745) on PPARD (PDB ID: 5U3Q). (**C**) MDT of β-Amyrone (PubChem ID: 612782) on PPARG (PDB ID: 3E00). (**D**) MDT of β-Amyrone (PubChem ID: 612782) on FABP3 (PDB ID: 5HZ9). (**E**) MDT of β-Amyrone (PubChem ID: 612782) on FABP4 (PDB ID: 3P6D). (**F**) MDT of β-Amyrone (PubChem ID: 612782) on NR1H3 (PDB ID: 2ACL).

It was observed that β-Amyrone had the highest affinity on five out of six target proteins: −16.1 kcal/mol on PPARA (PDB ID: 3SP6), −14.0 kcal/mol on PPARG (PDB ID: 3E00), −21.5 kcal/mol on FABP3 (PDB ID: 5HZ9), −13.2 kcal/mol on FABP4 (PDB ID: 3P6D), and −15.4 kcal/mol on NR1H3 (PDB ID: 2ACL). Interestingly, the highest affinity on PPARD (PDB ID: 5U3Q) was β-Stigmasterol with −10.8 kcal/mol. The docking detail information is enlisted in Table 6. Additionally, MDT was performed to compare bioactives with positive controls (Table 7). The results of MDT suggested that β-Amyrone on PPARA (PDB ID: 3SP6), PPARG (PDB ID: 3E00), and NR1H3 (PDB ID: 2ACL) had better affinity than the current positive controls. Moreover, it has been shown that β-Stigmasterol on PPARD (PDB ID: 5U3Q) had greater affinity than Cardarine used as an anti-obesity drug. The other two target proteins were not positive controls compared with β-Amyrone. Collectively, both β-Amyrone and β-Stigmasterol of CS on obesity were potential ligands to activate the PPAR signaling pathway. Its complex figures are depicted in Figure 7.

### 3.8. MDT of 7 Target Proteins, 3 Key Bioactives, and 15 Positive Controls on PI3K-Akt1 Signaling Pathway

**Figure 8:**
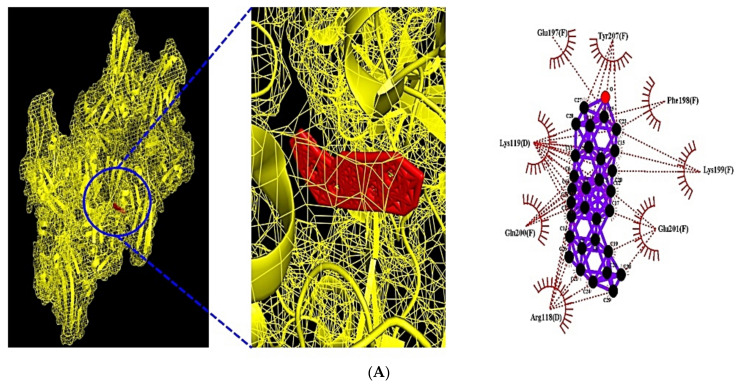

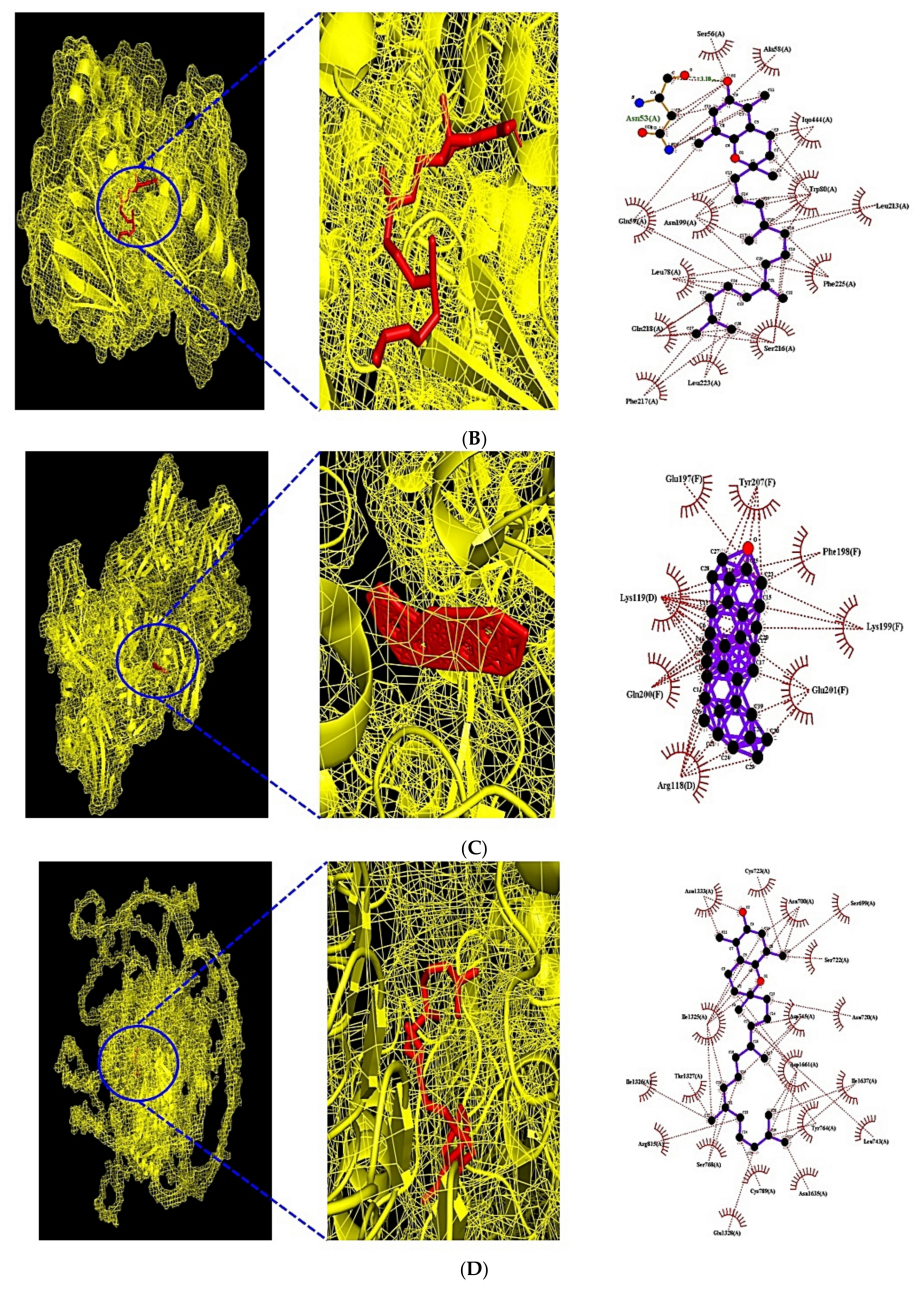
(**A**) MDT of Neotocopherol (PubChem ID: 86052) on AKT1 (PDB ID: 3O96). (**B**) MDT of Xanthosine (PubChem ID: 64959) on IL6 (PDB ID: 4NI9). (**C**) MDT of β-Amyrone (PubChem ID: 612782) on FGF2 (PDB ID: 1IIL). (**D**) MDT of Neotocopherol (PubChem ID: 86052) on PHLPP1.

Through MDT analysis, it was revealed that AKT1 (PDB ID: 3O96) was related to a sole bioactive: (1) Neotocopherol, IL6 (PDB ID: 4NI9) was associated with 3 bioactives: (1) Xanthosine, (2) 2-Ethylacridine, and (3) Linoleic acid, VEGFA (PDB ID: 3V2A) was connected to (1) Ethyl palmitate, (2) Ethyl heptadecanoate, (3) Ethyl stearate, (4) Ethyl linoleate, and (5) α-D-2-deoxyribose, PRKCA (PDB ID: 3IW4) was linked to 9 bioactives: (1) Ethyl palmitate, (2) Ethyl heptadecanoate, (3) Ethyl linoleate, (4) 2,4,4-Trimethylpentane-1,3-diyl bis(2-methylpropanoate), (5) Linoleic acid, (6) Ethyl stearate, (7) (Z)-9-Hexadecenal, (8) Palmitic acid, and (9) Oleic acid, FGF1 (PDB ID: 3OJ2) was related to 4 bioactives: (1) Sitostenone, (2) 24-epicampesterol, (3) Cytidine, and (4) α-D-2-deoxyribose, FGF2 (PDB ID: 1IIL) was associated with 7 bioactives: (1) β-Amyrone, (2) β-Stigmasterol, (3) Sitostenone, (4) 24-epicampesterol, (5) β-Sitosterol, (6) Cytidine, and (7) α-D-2-deoxyribose, and PHLPP1 was linked to a sole bioactive: (1) Neotocopherol. It was observed that Neotocopherol on AKT1 (PDB ID: 3O96), Xanthosine on IL6 (PDB ID: 4NI9), and β-Amyrone on FGF2 (PDB ID: 1IIL) had the highest affinity among bioactives from CS as well as better affinity than positive controls. The docking detail information is enlisted in Table 8. On the other hand, both Ethyl palmitate had the highest affinity on VEGFA (PDB ID: 3V2A), Sitostenone had the highest affinity on FGF1 (PDB ID: 3OJ2), and lower affinity than BAW2881 and Suramin, which were used as the positive controls, respectively. At present, it was observed that PHLPP1 was not enlisted in PDB, and had valid affinity with β-Amyrone (−7.2 kcal/mol). The detailed affinity value was exhibited in Table 9. The Autodock program was able to assemble active (Gibbs free energy of binding < −6.0 kcal/mol), suggesting that it had highly predictive affinity [37]. Comprehensively, Neotocopherol, Xanthosine, and β-Amyrone of CS on obesity were potential ligands to inhibit PI3K-Akt1 signaling pathway. Its complex figures are depicted in Figure 8.

## 4. Discussion

β-Amyrone, out of 36 bioactives from CS, was associated with the number of 6 target proteins on both the PPAR signaling pathway and the PI3K-Akt1 signaling pathway, considered as key signaling pathways of CS on obesity. Noticeably, it was unveiled that β-Amyrone (a triterpenoid derivative) on PPARA (PDB ID: 3SP6), PPARG (PDB ID: 3E00) and FGF2 (PDB ID: 1IIL) had better affinity than the positive controls. Likewise, the β-Stigmasterol on PPARD (PDB ID: 5U3Q) had better affinity than Cardarine, which is used as an anti-obesity drug. A report demonstrated that α,β-amyrin, as a triterpenoid derivative homologous to β-Amyrone, inhibits adipocyte differentiation by inactivating PPARG [38]. Another animal test showed that treatment of α,β-amyrin had a significant decrease in the level of blood glucose, serum triglyceride, and total cholesterol [39]. It implies that β-Amyrone might also be a potential ligand to exert an anti-adipogenic effect. A previous study showed that Stigmasterol significantly alleviated high-fat western-style fat (HFWD) induced fatty liver and metabolic disorders, including an increased level of hepatic total lipids, cholesterol, and triacylglycerols [40]. Furthermore, a report demonstrated that the activator of PPARA, PPARD, and PPARG is of great anti-obesity therapeutics due to the regulation of fat and gluconeogenesis [41]. Additionally, a report showed that the NR1H3 agonist makes good efficacy on the enhancement of reverse cholesterol transport, elevation of glucose uptake, and blocking of pro-inflammatory factors [42]. Additionally, Neotocopherol related directly to AKT1, considered as a hub target, had better affinity than two positive controls (AT13148, Afuresertib). There is a noticeable animal study indicating that knock-out of Akt1 elevates energy expenditure and, conversely, decreases the body weight of mice [43]. Another research shows that Akt1 null mice improved their insulin sensitivity and, thereby, elevated insulin secretion [44]. It could be speculated that the inhibitor of Akt1 might play a significant role to attenuate metabolic disorders, including obesity. The Vascular Endothelial Growth Factor A (VEGFA) is overexpressed in obese subjects while inhibitors of VEGF induced anti-proliferation of adipocytes induces weight loss [45,46]. The Fibroblast Growth Factor 2 (FGF2) is elevated in the context of obesity, the disruption of which leads to an increase of thermogenesis with higher energy expenditure and stable lipid maintenance [47,48]. It implies that the inhibitors of VEGFA and FGF2 might be potential ligands against obesity. The STB networks exhibited that the therapeutic effect of CS on obesity was directly associated with 27 bioactives. The KEGG pathway enrichment analysis of 27 bioactives shows that 12 signaling pathways were related to the occurrence and development of obesity, suggesting that these signaling pathways might be the pharmacological mechanisms of ABBR against obesity. The relationships of 12 signaling pathway with obesity were shortly discussed as follows. Advanced Glycation End Product-Receptor for Advanced Glycation End Product (AGE-RAGE) signaling pathway in diabetic complications: the AGE-RAGE signaling pathway influences the oxidative stress related to a diabetic complication, the inhibition of which is a therapeutic strategy for obesity [49,50]. Thyroid hormone signaling pathway: The elevated thyroid hormone levels attenuate the sensitivity of insulin to dampen hepatic glucose production and accelerates the glucose uptake in muscle cells [51]. It has been implicated that excessive thyroid hormone level leads to metabolic disorders, including obesity. Prolactin signaling pathway: It has been documented that prolactin level is increased in obese (17.75 ± 9.15 μg/L) subjects by comparison with subjects of normal weight (13.57 ± 9.03 μg/L) [52]. Estrogen signaling pathway: There is an observational outcome that estrogens play a crucial role in the occurrence of progression of female obesity, primarily via thyroid dysfunction and control of the hypothalamus [53]. Vascular endothelial growth factor (VEGF) signaling pathway: A report shows that inactivation of VEGF enhances the insulin sensitivity in high-fat-diet mice, which is an efficient approach to ameliorate obesity [54]. Phosphoinositide 3-Kinase–Protein Kinase B (PI3K-Akt) signaling pathway: A report demonstrated that inactivation of PI3K alleviates morbid overweight in obese mice and monkeys, indicating that the inhibitors did not induce drug resistance and adverse effects [55]. Additionally, alliin (40 μg/mL) as an inhibitor of Akt, inhibits adipogenesis by downregulating Akt [56]. Hypoxia Inducible Factor-1 (HIF-1) signaling pathway: The attenuation of HIF1-α alleviates glucose intolerance caused by obesity through diminishing Glucagon-Like Peptide-1 (GLP-1) [57]. Cyclic Adenosine MonoPhosphate (cAMP) signaling pathway: the elevation of cAMP level is linked to adipocyte differentiation as a negative factor of severe overweight, berberine known as cAMP inhibitor alleviates anti-obesity by lowering blood glucose, lipid, and body weight [58]. Repressor activator protein 1 (Rap1) signaling pathway: from two groups of mice fed a high fat diet, mice with functional Rap1 gain weight, in contrast, mice that deleted Rap1 remarkably reduced their body weight [59]. Renin-Angiotensin System (RAS) signaling pathway: a research shows that erucin is a bioactive compound isolated from broccoli, known as a Ras inhibitor, and has potent anti-obesity efficacy by inhibiting adipogenesis of 3T3-L1 cell line [60]. Mitogen-Activated Protein Kinase (MAPK) signaling pathway: MAPK, also known as ERK, the inhibition of which is a significant target to alleviate obesity via inhibiting adipogenic differentiation on MAPK signaling pathway [61]. Another research demonstrated that wedelolactone with inhibitory effect on MAPK signaling pathway ablates the adipocyte differentiation [62]. Peroxisome proliferator-activated receptor (PPAR) signaling pathway: a report demonstrated that PPAR activator is therapeutic strategy to alleviate obesity via burning fat brown adipose tissue (BAT), thereby diminishing the fat overload [63].

**Figure 9:**
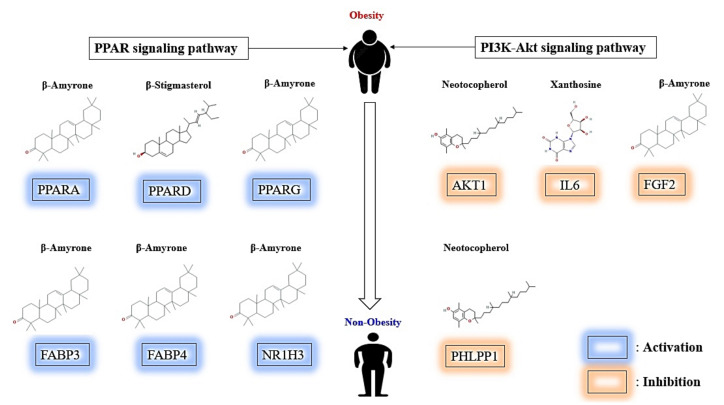
Summary representation of key findings in the study.

Besides, our study provided that 11 out 12 signaling pathways associated with AKT1 might have inhibitory effects for the alleviation of obesity, including PI3K-Akt signaling pathway. In contrast, PPAR signaling pathway of CS on obesity is a sole activator mechanism, not related to AKT1. According to a bubble chart, PPI, and STB networks results, we identified 2 signaling pathways, 13 targets, and 27 bioactives, and thus MDT verified that 4 bioactives (β-Amyrone, β-Stigmasterol, Neotocopherol, and Xanthosine) among 27 bioactives could stably bind to the targets, indicating that CS might activate the PPAR signaling pathway, and inactivate PI3K-Akt signaling pathway. Moreover, the final 4 bioactives have better stable affinity than the positive controls. To sum things up, we adopted 2 key signaling pathways (PPAR signaling pathway, PI3K-Akt signaling pathway), 10 targets (PPARA, PPARD, PPARG, FABP3, FABP4, NR1H3, AKT1, IL6, FGF2, and PHLPP1), and 4 bioactives (β-Amyrone, β-Stigmasterol, Neotocopherol, and Xanthosine) (see Figure 9). We removed three complexes (VEGFA-Ethyl palmitate, PRKCA- Ethyl palmitate, and FGF1-Sitostenone) with lower affinity than the positive controls. Hence, in the viewpoint of network pharmacology, this research elucidates promising signaling pathways, targets, and bioactives of CS against obesity, supporting a pharmacological basis for additional experimental validation.

## 5. Conclusions

Overall, this study demonstrated the potential signaling pathways, targets, and bioactives in treating obesity based on network pharmacology analysis. We identified 2 key signaling pathways (PPAR signaling pathway, PI3K-Akt signaling pathway), 13 targets (PPARA, PPARD, PPARG, FABP3, FABP4, NR1H3, AKT1, IL6, VEGFA, PRKCA, FGF1, FGF2, and PHLPP1), and 4 bioactives (β-Amyrone, β-Stigmasterol, Neotocopherol, and Xanthosine) of CS against obesity. A total of 10 out of 13 targets have better affinity or valid value in comparison with the positive controls: PPARA, PPARD, PPARG, FABP3, FABP4, NR1H3, AKT1, IL6, FGF2, and PHLPP1. The AKT1 with the highest degree value was considered as the uppermost target, Neotocopherol was a critical bioactive that was bound most stably to AKT1. Notably, β-Amyrone as an activator could dock well with PPARA, PPARG, FABP3, FABP4, NR1H3 on the PPAR signaling pathway, in contrast, β-Amyrone as an inhibitor could dock stably with FGF2 on the PI3K-Akt signaling pathway. This study shows that β-Amyrone of CS might have dual-efficacy to alleviate obesity. To conclude, we described the therapeutic evidence to expound key signaling pathways, targets, and bioactives of CS against obesity. However, there are still limitations to our analysis, which needs to be further improved, through either in vitro or in vivo. Last but not least, our analysis did not consider the expression of the target gene practically after treating the selected compounds, which should be implemented in the future.

## Figures and Tables

**Table 1:** A list of the detected 36 bioactives from CS through GC-MS.

No.	Compounds	PubChem ID	RT (mins)	Area (%)	Taxonomic Compound Classification
1	Ethylamine	6341	3.625	2.37	Amines
2	cis-2,3-Epoxybutane	92162	4.097	2.77	Epoxides
3	5-Hydroxymethylfurfural	237332	4.683	8.2	Carbonyl compounds
4	Mannitan	10909888	5.135	0.36	Tetrahydrofurans
5	5-Aminovaleric acid	138	5.164	0.67	Amino acids, peptides, and analogues
6	Nitrous acid, 1-methylpropyl ester	13544	5.270	1.18	Organic nitroso compounds
7	Formicin	69365	5.356, 5.481	1.76	Carboxylic acid derivatives
8	Diethyl acetal	7765	5.818	1.39	Ethers
9	Xanthosine	64959	6.337	7.91	Purine nucleosides
10	Cytidine	6175	6.395	6.33	Pyrimidine nucleosides
11	2,4,4-Trimethylpentane-1,3-diyl bis(2-methylpropanoate)	93439	6.606	1.33	Dicarboxylic acids and derivatives
12	α-D-2-deoxyribose	441475	7.116	3.4	Oxanes
13	2R,3S-9-[1,3,4-Trihydroxy-2-butoxymethyl]guanine	135789714	7.193	3.51	Purines and purine derivatives
14	Palmitic acid	985	8.250, 8.616	5.2	Fatty acids and conjugates
15	Ethyl palmitate	12366	8.318	3.35	Fatty acid esters
16	Linoleic acid	5280450	8.914	1.85	Lineolic acids and derivatives
17	Ethyl linoleate	5282184	8.952	4.24	Lineolic acids and derivatives
18	Ethyl stearate	8122	9.058	1.41	Fatty acid esters
19	Estradiol, 3-deoxy	537293	9.414	0.74	Estrane steroids
20	Oleic Acid	445639	9.645	0.59	Fatty acids and conjugates
21	Ethyl isovalerate	7945	9.731	0.76	Fatty acid esters
22	Eicosane	8222	10.058, 10.721, 11.529	2.71	Alkanes
23	(Z)-9-Hexadecenal	5364643	10.164	1.61	Fatty aldehydes
24	Heneicosanoic acid, 2,4-dimethyl-, methyl ester	560463	10.366	1.29	Fatty acid esters
25	7-Pentadecyne	549063	10.789	1.32	Acetylenes
26	Ethyl heptadecanoate	26397	11.096	0.9	Fatty acid esters
27	Squalene	638072	11.193	0.76	Triterpenoids
28	1,3-Dioxolane, 4-ethyl-5-octyl-2,2-bis(trifluoromethyl)-, trans-	91694992	12.423	0.24	Ethers
29	Neotocopherol	86052	12.462	0.74	1-hydroxy-4-unsubstituted benzenoids
30	N,2-diaminopropane	7210	12.731, 14.356	1.02	1-hydroxy-4-unsubstituted benzenoids
31	24-epicampesterol	5283637	13.895	2.85	Ergostane steroids
32	β-Stigmasterol	6432745	14.116	5.32	Stigmastanes and derivatives
33	β-Sitosterol	222284	14.721	12.41	Triterpenoids
34	β-Amyrone	612782	14.895, 15.385	6.95	Triterpenoids
35	Sitostenone	5484202	16.087	1.64	Stigmastanes and derivatives
36	2-Ethylacridine	610161	18.308	0.91	Benzoquinolines

**Table 2:** Physicochemical properties of 36 bioactives for Lipinski’s rule, bioavailability, and cell membrane permeability.

No.	Compounds	Lipinski Rules				Lipinski’s Violations	Bioavailability Score	TPSA(Å^2^)
		MW	HBA	HBD	MLog P			
		<500	<10	≤5	≤4.15	≤1	>0.1	<140
1	Ethylamine	45.08	1	1	−0.23	0	0.55	26.02
2	cis-2,3-Epoxybutane	72.11	1	0	0.35	0	0.55	12.53
3	5-Hydroxymethylfurfural	126.11	3	1	−1.06	0	0.55	50.44
4	Mannitan	164.16	5	4	−2.35	0	0.55	90.15
5	5-Aminovaleric acid	117.15	3	2	0.01	0	0.55	63.32
6	Nitrous acid, 1-methylpropyl ester	103.12	3	0	0.42	0	0.55	38.66
7	Formicin	89.09	2	2	−0.85	0	0.55	49.33
8	Diethyl acetal	118.17	2	0	1.01	0	0.55	18.46
9	Xanthosine	284.23	7	5	−2.30	0	0.55	153.46
10	Cytidine	243.22	6	4	−2.29	0	0.55	130.83
11	2,4,4-Trimethylpentane-1,3-diyl bis(2-methylpropanoate)	286.41	4	0	3.17	0	0.55	52.60
12	α-D-2-deoxyribose	134.13	4	3	−1.49	0	0.55	69.92
13	2R,3S-9-[1,3,4-Trihydroxy-2-butoxymethyl] guanine	285.26	7	5	−2.76	0	0.55	159.51
14	Palmitic acid	256.42	2	1	4.19	1	0.85	37.30
15	Ethyl palmitate	284.48	2	0	4.67	1	0.55	26.30
16	Linoleic acid	280.45	2	1	4.47	1	0.85	37.30
17	Ethyl linoleate	308.50	2	0	4.93	1	0.55	26.30
18	Ethyl stearate	312.53	2	0	5.13	1	0.55	26.30
19	Estradiol, 3-deoxy	256.38	1	1	4.19	1	0.55	20.23
20	Oleic Acid	282.46	2	1	4.57	1	0.85	37.30
21	Ethyl isovalerate	130.18	2	0	1.63	0	0.55	26.30
22	Eicosane	282.55	0	0	7.38	1	0.55	0.00
23	(Z)-9-Hexadecenal	238.41	1	0	4.20	1	0.55	17.07
24	Heneicosanoic acid, 2,4-dimethyl-, methyl ester	368.64	2	0	6.00	1	0.55	26.30
25	7-Pentadecyne	208.38	0	0	6.04	1	0.55	0.00
26	Ethyl heptadecanoate	298.50	2	0	4.91	1	0.55	26.30
27	Squalene	410.72	0	0	7.93	1	0.55	0.00
28	1,3-Dioxolane, 4-ethyl-5-octyl-2,2-bis(trifluoromethyl)-, trans-	350.34	8	0	4.02	0	0.55	18.46
29	Neotocopherol	416.68	2	1	5.94	1	0.55	29.46
30	N,2-diaminopropane	282.34	5	4	1.81	0	0.55	65.18
31	24-epicampesterol	400.68	1	1	6.54	1	0.55	20.23
32	β-Stigmasterol	412.69	1	1	6.62	1	0.55	20.23
33	β-Sitosterol	414.71	1	1	6.73	1	0.55	20.23
34	β-Amyrone	424.70	1	0	6.82	1	0.55	17.07
35	Sitostenone	412.69	1	0	6.62	1	0.55	17.07
36	2-Ethylacridine	207.27	1	0	3.58	0	0.55	12.89

**Table 3:** The degree value of 79 targets in PPI.

No.	Target	Degree of Value	No.	Target	Degree of Value
1	AKT1	43	41	NR1I2	7
2	IL6	39	42	ADORA3	6
3	GAPDH	33	43	ALOX15	6
4	PPARG	29	44	EIF4E	6
5	VEGFA	29	45	FFAR4	6
6	ESR1	25	46	GLI1	6
7	PPARA	23	47	HSD11B2	6
8	AR	19	48	NPC1L1	6
9	CYP19A1	19	49	NR1H2	6
10	CNR1	16	50	RAC1	6
11	FGF2	15	51	SERPINA6	6
12	NR3C1	15	52	VDR	6
13	CDC42	12	53	PPARD	6
14	CYP17A1	12	54	CNR2	5
15	ESR2	12	55	FABP3	5
16	PRKCA	12	56	GPBAR1	5
17	TRPV1	12	57	ESRRB	4
18	SREBF2	11	58	NAAA	4
19	NR1H4	10	59	PDK1	4
20	SHBG	10	60	PDCD4	4
21	SRD5A1	10	61	ALOX12	3
22	FABP4	9	62	CES1	3
23	GPER1	9	63	CPB2	3
24	HSD17B1	9	64	ENPP2	3
25	HSPA5	9	65	MGEA5	3
26	NR1H3	9	66	MTNR1B	3
27	NR3C2	9	67	IMPDH2	3
28	STS	9	68	PHLPP1	3
29	ADORA1	8	69	ACP1	2
30	ADORA2A	8	70	CES2	2
31	ADORA2B	8	71	EHMT1	2
32	AKR1C3	8	72	FDFT1	2
33	ESRRA	8	73	RORC	2
34	F2	8	74	SLC5A2	2
35	FAAH	8	75	TBXA2R	2
36	PLA2G1B	8	76	ADCY10	1
37	ADA	7	77	ENPEP	1
38	FGF1	7	78	NPEPPS	1
39	G6PD	7	79	SLC22A2	1
40	MMP3	7			

**Table 4:** Targets in 12 signaling pathways enrichment associated with obesity.

KEGG ID	Targets	False Discovery Rate
hsa03320:PPAR signaling pathway	PPARA, PPARD, PPARG, FABP3, FABP4, NR1H3	0.0001200
hsa04370:VEGF signaling pathway	AKT1, VEGFA, PRKCA, CDC42	0.0049000
hsa04917:Prolactin signaling pathway	AKT1, ESR1, ESR2, CYP17A1	0.0080000
hsa04066:HIF-1 signaling pathway	AKT1, IL6, GAPDH, VEGFA, PRKCA, PDK1	0.0006900
hsa04933:AGE-RAGE signaling pathway in diabetic complications	AKT1, IL6, VEGFA, PRKCA, CDC42	0.0035000
hsa04015:Rap1 signaling pathway	AKT1, VEGFA, CDC42, PRKCA, FGF1, FGF2, CNR1, ADRA2A, ADORA2B	0.0000376
hsa04919:Thyroid hormone signaling pathway	AKT1, PRKCA, ESR1, THRA	0.0337000
hsa04014:Ras signaling pathway	AKT1, VEGFA, CDC42, PRKCA, FGF1, FGF2	0.0031000
hsa04915:Estrogen signaling pathway	AKT1, ESR1, ESR2, GPER1	0.0480000
hsa04024:cAMP signaling pathway	AKT1, PPARA, ADORA2A, GLI1, ADORA1, ADCY10	0.0089000
hsa04010:MAPK signaling pathway	AKT1, VEGFA, CDC42, PRKCA, FGF1, FGF2	0.0328000
hsa04151:PI3K-Akt signaling pathway	AKT1, IL6, VEGFA, PRKCA, FGF1, FGF2, PHLPP1	0.0194000

**Table 5:** The degree value of 28 targets in STB.

No.	Target	Degree of Value	No.	Target	Degree of Value
1	AKT1	11	15	PPARG	1
2	PRKCA	8	16	FABP4	1
3	VEGFA	7	17	CYP17A1	1
4	CDC42	5	18	GAPDH	1
5	FGF1	4	19	PDK1	1
6	FGF2	4	20	CNR1	1
7	ESR1	3	21	ADORA2B	1
8	IL6	3	22	THRA	1
9	PPARA	2	23	PLA2G1B	1
10	ESR2	2	24	GPER1	1
11	ADORA2A	2	25	GLI1	1
12	FABP3	1	26	ADORA1	1
13	NR1H3	1	27	ADCY10	1
14	PPARD	1	28	PHLPP1	1

**Table 6:** Binding energy and interactions of potential bioactives on the PPAR signaling pathway.

				Grid Box		Hydrogen Bond Interactions	Hydrophobic Interactions
Protein	Ligand	PubChem ID	Binding Energy (kcal/mol)	Center	Dimension	Amino Acid Residue	Amino Acid Residue
PPARA (PDB ID: 3SP6)	(*) β-Amyrone	612782	−16.1	x = 8.006	size_x = 40	N/A	Tyr334, Ala333, Val324
				y = −0.459	size_y = 40		Met320, Phe218, Met220
				z = 23.392	size_z = 40		Glu286, Val332, Asn219
							Thr279
	Squalene	638072	−6.0	x = 8.006	size_x = 40	N/A	Tyr334, Asn336, Ala333
				y = −0.459	size_y = 40		Thr279, Leu254, Val332
				z = 23.392	size_z = 40		Ile241, Glu251, Ala250
							Cys275, Cys278, Val255
	Ethyl palmitate	12366	−6.0	x = 8.006	size_x = 40	N/A	Met320, Leu321, Val324
				y = −0.459	size_y = 40		Leu331, Val332, Ala333
				z = 23.392	size_z = 40		Thr279, Tyr334, Asn219
							Thr283, Ile317
	Heneicosanoic acid, 2,4-dimethyl-, methyl ester	560463	−5.8	x = 8.006	size_x = 40	N/A	Lys257, Val255, His274
				y = −0.459	size_y = 40		Leu254, Ala250, Glu251
				z = 23.392	size_z = 40		Ala333, Cys275, Cys278
							Leu258
	Oleic Acid	445639	−5.3	x = 8.006	size_x = 40	N/A	Leu254, Val255, Ala250
				y = −0.459	size_y = 40		Ala333, Asn219, Thr283
				z = 23.392	size_z = 40		Met320, Leu321, Val324
							Ile317, Thr279, Tyr334
							Cys275, Glu251
	Ethyl linoleate	5282184	−5.0	x = 8.006	size_x = 40	N/A	Glu282, Tyr334, Thr279
				y = −0.459	size_y = 40		Ala333, Glu251, Leu254
				z = 23.392	size_z = 40		Cys275, Ala250, Val255
							Cys278, Val281
	Palmitic acid	985	−4.9	x = 8.006	size_x = 40	N/A	Val332, Ile241, Ala333
				y = −0.459	size_y = 40		Thr279, Val255, Tyr334
				z = 23.392	size_z = 40		Leu258, Cys275, Ala250
							Leu254, Glu251
	Linoleic acid	5280450	−4.9	x = 8.006	size_x = 40	Ser323, Tyr214	Asn221, Met320, Val324
				y = −0.459	size_y = 40		Met320, Asn219, Tyr334
				z = 23.392	size_z = 40		Thr279, Leu331, Leu321
							Thr283, Ile317
	(Z)-9-Hexadecenal	5364643	−3.8	x = 8.006	size_x = 40	Thr307	Asn303, Lys310, Tyr311
				y = −0.459	size_y = 40		Pro389, Asp466, Ser688
				z = 23.392	size_z = 40		Val306
PPARD (PDB ID: 5U3Q)	(*) β-Stigmasterol	6432745	−10.8	x = 39.265	size_x = 40	N/A	Ser271, Glu262, Ser271
				y = −18.736	size_y = 40		Pro268, Ser266, Lys265
				z = 119.392	size_z = 40		
	β-Sitosterol	222284	−7.1	x = 39.265	size_x = 40	Arg407, Glu288	Asp439, Tyr284, Pro362
				y = −18.736	size_y = 40		Met440, Val410, Thr411
				z = 119.392	size_z = 40		
	Heneicosanoic acid, 2,4-dimethyl-, methyl ester	560463	−5.9	x = 39.265	size_x = 40	N/A	Met440, Thr411, Val410
				y = −18.736	size_y = 40		Tyr441, Pro362, Asp360
				z = 119.392	size_z = 40		Arg361, Arg407, Tyr284
	Ethyl linoleate	5282184	−5.6	x = 39.265	size_x = 40	N/A	Met440, Tyr441, Asp439
				y = −18.736	size_y = 40		Pro362, Tyr284, Arg361
				z = 119.392	size_z = 40		Val410, Thr411
	Linoleic acid	5280450	−5.2	x = 39.265	size_x = 40	N/A	Val410, Arg407, Met440
				y = −18.736	size_y = 40		Asp439, Thr411, Tyr411
				z = 119.392	size_z = 40		Tyr284, Asp360, Pro362
							Arg361, Glu288
	Oleic Acid	445639	−4.9	x = 39.265	size_x = 40	N/A	Asp360, Pro362, Tyr284
				y = −18.736	size_y = 40		Val410, Met440, Tyr441
				z = 119.392	size_z = 40		Thr411
	Palmitic acid	985	−4.6	x = 39.265	size_x = 40	N/A	Tyr441, Pro362, Arg361
				y = −18.736	size_y = 40		Val410, Tyr284, Glu288
				z = 119.392	size_z = 40		Met440, Thr411, Ala414
							Arg407
	(Z)-9-Hexadecenal	5364643	−3.7	x = 39.265	size_x = 40	Arg361	Thr411, Arg407, Pro362
				y = −18.736	size_y = 40		Asp439, Met440
				z = 119.392	size_z = 40		
PPARG (PDB ID: 3E00)	(*) β-Amyrone	612782	−14.0	x = 2.075	size_x = 40	N/A	Glu343, Glu295, Ile326
				y = 31.910	size_y = 40		Ile296, Ala292, Phe226
				z = 18.503	size_z = 40		Met329, Leu333, Pro227
							Leu228
	Ethyl linoleate	5282184	−5.9	x = 2.075	size_x = 40	N/A	Ile296, Met329, Ile326
				y = 31.910	size_y = 40		Leu228, Leu333, Ala292
				z = 18.503	size_z = 40		Arg288, Phe226, Glu295
	Linoleic acid	5280450	−5.3	x = 2.075	size_x = 40	Thr162, Leu167	Arg202, Tyr192, Asp166
				y = 31.910	size_y = 40		Lys336, Glu369, Val372
				z = 18.503	size_z = 40		Arg350, Glu351, Gln193
							Lys354
	Oleic Acid	445639	−5.1	x = 2.075	size_x = 40	Asp441, Asn377	Arg426, Lys381, Asp379
				y = 31.910	size_y = 40		Phe370, Lys373, Glu448
				z = 18.503	size_z = 40		Ile445, Pro366, Glu369
							Gln444
	Palmitic acid	985	−5.1	x = 2.075	size_x = 40	Glu291, Arg288	Glu343, Leu333, Leu330
				y = 31.910	size_y = 40		Leu228, Met329, Ala292
				z = 18.503	size_z = 40		Ile326, Glu295
	(Z)-9-Hexadecenal	5364643	−5.0	x = 2.075	size_x = 40	N/A	Met329, Leu333, Ser332
				y = 31.910	size_y = 40		Leu228, Arg288, Glu295
				z = 18.503	size_z = 40		Glu343, Ala292
FABP3 (PDB ID: 5HZ9)	(*) β-Amyrone	612782	−21.5	x = −1.215	size_x = 40	N/A	Phe28, Gln32, Lys32
				y = 46.730	size_y = 40		Thr57, Ala29
				z = −15.099	size_z = 40		
	Heneicosanoic acid, 2,4-dimethyl-, methyl ester	560463	−8.8	x = −1.215	size_x = 40	N/A	Glu27, Phe28, Gly25
				y = 46.730	size_y = 40		Ala29, Lys22, Gln32
				z = −15.099	size_z = 40		
	Eicosane	8222	−8.6	x = −1.215	size_x = 40	N/A	Lys22, Thr57, Gln32
				y = 46.730	size_y = 40		Gly25, Phe28, Ala29
				z = −15.099	size_z = 40		
	Ethyl stearate	8122	−8.3	x = −1.215	size_x = 40	N/A	Phe58, Gln32, Gly25
				y = 46.730	size_y = 40		Phe28
				z = −15.099	size_z = 40		
	Ethyl heptadecanoate	26397	−8.3	x = −1.215	size_x = 40	N/A	Gly27, Gln32, Ala29
				y = 46.730	size_y = 40		Phe28
				z = −15.099	size_z = 40		
	Ethyl linoleate	5282184	−8.2	x = −1.215	size_x = 40	N/A	Lys22, Ala29, Phe28
				y = 46.730	size_y = 40		Gly25, Gln32
				z = −15.099	size_z = 40		
	Ethyl palmitate	12366	−8.2	x = −1.215	size_x = 40	N/A	Ala29, Gln32, Phe28
				y = 46.730	size_y = 40		Gly25, Gly27, Lys22
				z = −15.099	size_z = 40		
	Linoleic acid	5280450	−7.4	x = −1.215	size_x = 40	Lys22	Ala29, Gln32, Phe28
				y = 46.730	size_y = 40		Gly25, Gly27
				z = −15.099	size_z = 40		
	(Z)-9-Hexadecenal	5364643	−6.9	x = −1.215	size_x = 40	N/A	Gly27, Gly25, Gln32
				y = 46.730	size_y = 40		Ala29, Phe28, Ala29
				z = −15.099	size_z = 40		
	Oleic Acid	445639	−6.9	x = −1.215	size_x = 40	N/A	Phe28, Gly27, Ala29
				y = 46.730	size_y = 40		Gln32
				z = −15.099	size_z = 40		
	Palmitic acid	985	−6.5	x = −1.215	size_x = 40	N/A	Val33, Gln32, Ala29
				y = 46.730	size_y = 40		Phe58, Lys22, Thr57
				z = −15.099	size_z = 40		
	5-Aminovaleric acid	138	−5.1	x = −1.215	size_x = 40	Ala29, Phe28, Val26	Gly25, Gly27, Lys22
				y = 46.730	size_y = 40		Phe28
				z = −15.099	size_z = 40		
FABP4 (PDB ID: 3P6D)	(*) β-Amyrone	612782	−13.2	x = 7.693	size_x = 40	N/A	Val90, Lys107, Glu109
				y = 9.921	size_y = 40		Glu116, Val114, Lys105
				z = 14.698	size_z = 40		
	Heneicosanoic acid, 2,4-dimethyl-, methyl ester	560463	−5.6	x = 7.693	size_x = 40	Leu86	Leu66, Ile49, Asp47
				y = 9.921	size_y = 40		Cys1, Ser1, Met0
				z = 14.698	size_z = 40		
	Ethyl stearate	8122	−5.5	x = 7.693	size_x = 40	Gly88	Asp87, Leu86, Asp47
				y = 9.921	size_y = 40		Leu66, Gly46, Ile49
				z = 14.698	size_z = 40		Val44, Ser1, Cys1
							Met0
	Ethyl palmitate	12366	−5.4	x = 7.693	size_x = 40	N/A	Ala29, Gln32, Phe28
				y = 9.921	size_y = 40		Gly25, Gly27, Lys22
				z = 14.698	size_z = 40		
	Ethyl heptadecanoate	26397	−5.3	x = 7.693	size_x = 40	Gly88	Asp87, Leu86, Asp47
				y = 9.921	size_y = 40		Ile49, Ser1, Leu66
				z = 14.698	size_z = 40		Cys1, Met0
	Ethyl linoleate	5282184	−5.2	x = 7.693	size_x = 40	Gly88	Asp87, Met0, Ile49
				y = 9.921	size_y = 40		Cys1, Gly46, Asp47
				z = 14.698	size_z = 40		Ser1, Leu66, Leu86
	5-Aminovaleric acid	138	−5.0	x = 7.693	size_x = 40	Arg106, Gln96, Glu72	Thr60, Ala75, Thr74
				y = 9.921	size_y = 40		
				z = 14.698	size_z = 40		
	Oleic Acid	445639	−5.0	x = 7.693	size_x = 40	Leu86	Gly88, Ile49, Val44
				y = 9.921	size_y = 40		Gly46, Asp47, Ile65
				z = 14.698	size_z = 40		Leu66, Asp87
	Linoleic acid	5280450	−4.9	x = 7.693	size_x = 40	Leu86	Thr85, Leu66, Asp47
				y = 9.921	size_y = 40		Cys1, Gly46, Ser1
				z = 14.698	size_z = 40		Met0
	Palmitic acid	985	−4.4	x = 7.693	size_x = 40	Glu72, Val80	Lys79, Asp71, Val73
				y = 9.921	size_y = 40		Glu61, Thr60
				z = 14.698	size_z = 40		
	(Z)-9-Hexadecenal	5364643	−4.0	x = 7.693	size_x = 40	N/A	Glu109, Val90, Lys105
				y = 9.921	size_y = 40		Lys107, Val114, Glu116
				z = 14.698	size_z = 40		
NR1H3 (PDB ID: 2ACL)	(*) β-Amyrone	612782	−15.4	x = 48.735	size_x = 40	N/A	Gln330, Ala325, Gly328
				y = 39.677	size_y = 40		Arg248, Arg245, Lys431
				z = 77.096	size_z = 40		Gln297, Leu294, Gln429
							Val298, Asp295, Leu329
	β-Stigmasterol	6432745	−11.1	x = 48.735	size_x = 40	N/A	Gln429, Arg248, Gly328
				y = 39.677	size_y = 40		Leu329, Gln330, Glu332
				z = 77.096	size_z = 40		Ile299, Val331, Arg302
							Val298, Asp295, Leu294
	β-Sitosterol	222284	−8.1	x = 48.735	size_x = 40	Asn385	Pro237, Glu388, Glu322
				y = 39.677	size_y = 40		Ala391, Lys395, Ala398
				z = 77.096	size_z = 40		Leu400, Glu394, Pro240
							Lys326, Ile238
	Estradiol, 3-deoxy	537293	−8.0	x = 48.735	size_x = 40	N/A	Pro240, Leu347, Ala343
				y = 39.677	size_y = 40		Asp379, Ser411, Arg404
				z = 77.096	size_z = 40		Met407, Lys408, Glu390
							Glu346
	Sitostenone	5484202	−7.7	x = 48.735	size_x = 40	Asn385	Glu388, Ala391, Lys395
				y = 39.677	size_y = 40		Pro240, Glu322, Glu394
				z = 77.096	size_z = 40		Leu400, Asp241, Trp236
							Pro242, Arg251, Lys326
							Ile238, Pro237
	24-epicampesterol	5283637	−7.7	x = 48.735	size_x = 40	Asn385	Glu388, Ala391, Lys395
				y = 39.677	size_y = 40		Glu394, Asp241, Pro242
				z = 77.096	size_z = 40		Pro240, Lys326, Ile238
							Glu322, Pro237
	Ethyl linoleate	5282184	−6.0	x = 48.735	size_x = 40	N/A	Gln330, Lys381, Ile299
				y = 39.677	size_y = 40		Asp295, Leu294, Lys431
				z = 77.096	size_z = 40		Arg248, Gln429, Val298
							Arg302, Gly382
	Linoleic acid	5280450	−5.2	x = 48.735	size_x = 40	Ser411	Leu347, Arg404, Pro378
				y = 39.677	size_y = 40		Asp379, Ala387, Pro386
				z = 77.096	size_z = 40		Glu390, Pro240, Glu346
							Lys408, Ala343
	Oleic Acid	445639	−4.9	x = 48.735	size_x = 40	N/A	Arg404, Glu390, Lys408
				y = 39.677	size_y = 40		Arg342, Glu339, Pro386
				z = 77.096	size_z = 40		Pro240, Glu346, Tyr397
							Met407

(*): The most stable bioactive on a target.

**Table 7:** Binding energy and interactions of potential bioactives on the PI3K-Akt signaling pathway.

				Grid Box		Hydrogen Bond Interactions	Hydrophobic Interactions
Protein	Ligand	PubChem ID	Binding Energy (kcal/mol)	Center	Dimension	Amino Acid Residue	Amino Acid Residue
AKT1 (PDB ID: 3O96)	(*) Neotocopherol	86052	−6.6	x = 6.313	size_x = 40	Asn53	Ser56, Ala58, Trp80
				y = −7.926	size_y = 40		Leu213, Phe225, Ser216
				z = 17.198	size_z = 40		Leu223, Phe217, Gln218
							Leu78, Gln59, Asn199
IL6 (PDB ID: 4NI9)	(*) Xanthosine	64959	−7.4	x = 11.213	size_x = 40	Ser37	Asp34, Ala38
				y = 33.474	size_y = 40		
				z = 11.162	size_z = 40		
	2-Ethylacridine	610161	−6.7	x = 11.213	size_x = 40	N/A	Asp34, Gly35, Tyr31
				y = 33.474	size_y = 40		Gln111
				z = 11.162	size_z = 40		
	Linoleic acid	5280450	−5.0	x = 11.213	size_x = 40	Lys39	Glu81, Pro80, Ser168
				y = 33.474	size_y = 40		Phe83, Glu105, Leu104
				z = 11.162	size_z = 40		Gln166, Lys103, Glu165
							Pro40, Ile106
VEGFA (PDB ID: 3V2A)	(*) Ethyl palmitate	12366	−6.4	x = 38.009	size_x = 40	N/A	Gly196, Lys48, Ile215
				y = −10.962	size_y = 40		Ile80, Met81, Ile91
				z = 12.171	size_z = 40		Gln79, His133, Pro49
							Tyr165
	Ethyl heptadecanoate	26397	−5.1	x = 38.009	size_x = 40	N/A	Pro40, Asp276, Phe36
				y = −10.962	size_y = 40		Lys48, Phe47, Ile46
				z = 12.171	size_z = 40		Lys286, Asp34
	Ethyl stearate	8122	−5.0	x = 38.009	size_x = 40	N/A	Gln87, Gly88, Tyr137
				y = −10.962	size_y = 40		Ile138, Lys144, Val146
				z = 12.171	size_z = 40		Ser189, Thr45, Thr139
							His86
	Ethyl linoleate	5282184	−4.9	x = 38.009	size_x = 40	N/A	Pro40, Asp276, Asp34
				y = −10.962	size_y = 40		Phe47, Lys48, Asn253
				z = 12.171	size_z = 40		Ile46, Lys286, Phe36
	α-D-2-deoxyribose	441475	−4.2	x = 38.009	size_x = 40	Gly255, Ser310, Gly312	Glu44, Asp257, Lys84
				y = −10.962	size_y = 40		Pro85, Ser311
				z = 12.171	size_z = 40		
PRKCA (PDB ID: 3IW4)	(*) Ethyl palmitate	12366	−6.4	x = −14.059	size_x = 40	N/A	Gly196, Met197, Ile80
				y = 38.224	size_y = 40		Met81, Ile91, Gln79
				z = 32.319	size_z = 40		His133, Pro49, Lys48
							Tyr165, Ile215
	Ethyl heptadecanoate	26397	−6.2	x = −14.059	size_x = 40	Lys396	Leu393, Asn660, Gln402
				y = 38.224	size_y = 40		Pro666, Ile667, Glu474
				z = 32.319	size_z = 40		Lys478, Pro398, Val664
							Pro397, Arg608
	Ethyl linoleate	5282184	−6.2	x = −14.059	size_x = 40	Lys396	Asn660, Gln402, Pro666
				y = 38.224	size_y = 40		Val664, Glu418, His665
				z = 32.319	size_z = 40		Arg608, Lys478, Pro398
							Asp395, Leu393, Leu394
	2,4,4-Trimethylpentane-1,3-diyl bis(2-methylpropanoate)	93439	−6.0	x = −14.059	size_x = 40	Asp472, His476, Arg608	Ser670, Ile510, Met551
				y = 38.224	size_y = 40		Gln548, Glu609, Asp544
				z = 32.319	size_z = 40		Glu552, Ile667, Asn607
							Leu668, Glu474
	Linoleic acid	5280450	−5.4	x = −14.059	size_x = 40	Lys396	Leu393, Pro397, Asn660
				y = 38.224	size_y = 40		Leu394, Ser549, Gln662
				z = 32.319	size_z = 40		Gln548, His553, Glu552
							Val664, Pro398, Gln402
	Ethyl stearate	8122	−5.0	x = −14.059	size_x = 40	N/A	Arg275, Phe36, Asp34
				y = 38.224	size_y = 40		Lys48, Phe47, Ile46
				z = 32.319	size_z = 40		Lys286, Asp276, Pro40
	(Z)-9-Hexadecenal	5364643	−4.2	x = −14.059	size_x = 40	Asp395, Lys396	Gln402, Pro398, Val664
				y = 38.224	size_y = 40		Glu552, Gln662, Leu394
				z = 32.319	size_z = 40		
	Palmitic acid	985	−3.8	x = −14.059	size_x = 40	Phe47	Phe36, Lys286, Leu252
				y = 38.224	size_y = 40		Leu277, Asp276, Ile46
				z = 32.319	size_z = 40		Asn253, Ser50
	Oleic Acid	445639	−3.5	x = −14.059	size_x = 40	N/A	Thr145, Val146, Ile138
				y = 38.224	size_y = 40		His86, Leu313, Thr139
				z = 32.319	size_z = 40		Tyr137, Ser189, Lys144
FGF1 (PDB ID: 3OJ2)	(*) Sitostenone	5484202	−8.5	x = 9.051	size_x = 40	N/A	Arg203, Ser220, Val222
				y = 22.527	size_y = 40		Phe172, Ile257, Ser282
				z = −0.061	size_z = 40		Pro19, Tyr281, Ile204
							Ala260
	24-epicampesterol	5283637	−8.3	x = 9.051	size_x = 40	Ile204	Arg203, Val222, Tyr281
				y = 22.527	size_y = 40		Pro19, Ile257, Ser220
				z = −0.061	size_z = 40		Ala260
	Cytidine	6175	−6.7	x = 9.051	size_x = 40	Asn350, Arg255, Gln351	Asn107, Asn173, Phe172
				y = 22.527	size_y = 40		Leu258, Ser220, Ala349
				z = −0.061	size_z = 40		Thr174
	α-D-2-deoxyribose	441475	−5.2	x = 9.051	size_x = 40	Gln348, Asn350, Thr174	Ala349, Phe172
				y = 22.527	size_y = 40	Asn173, Asn107, Arg255	
				z = −0.061	size_z = 40		
FGF2 (PDB ID: 1IIL)	(*) β-Amyrone	612782	−14.4	x = 26.785	size_x = 40	N/A	Glu197, Tyr207, Phe198
				y = 14.360	size_y = 40		Lys199, Glu201, Arg118
				z = −1.182	size_z = 40		Gln200, Lys119
	β-Stigmasterol	6432745	−10.9	x = 26.785	size_x = 40	Arg118	Glu201, Asp99, Gln200
				y = 14.360	size_y = 40		Tyr207, Val209, Lys119
				z = −1.182	size_z = 40		
	Sitostenone	5484202	−7.7	x = 26.785	size_x = 40	His254	Ala172, Val222, Ile204
				y = 14.360	size_y = 40		Ser220, Leu258, Gln259
				z = −1.182	size_z = 40		Ala260, Ile257
	24-epicampesterol	5283637	−7.7	x = 26.785	size_x = 40	Ser137	Thr139, Trp123, Lys13
				y = 14.360	size_y = 40		Leu312, Asp336, Tyr340
				z = −1.182	size_z = 40		Ile329, Tyr328, Leu327
							Ser122, Glu323
	β-Sitosterol	222284	−7.2	x = 26.785	size_x = 40	Asp336, Tyr340	Ile329, Leu327, Ser122
				y = 14.360	size_y = 40		Thr319, Lys313, Leu312
				z = −1.182	size_z = 40		Lys292
	Cytidine	6175	−6.4	x = 26.785	size_x = 40	Tyr328, Lys313, Thr319	Pro141, Glu323
				y = 14.360	size_y = 40	Asn318, Ser122	
				z = −1.182	size_z = 40		
	α-D-2-deoxyribose	441475	−4.6	x = 26.785	size_x = 40	Arg255, Phe352	Ala172, Thr174, Ser351
				y = 14.360	size_y = 40		Asn173, His353
				z = −1.182	size_z = 40		
PHLPP1 (not available in the PDB)	(*) Neotocopherol	86052	−7.2	x = 26.785	size_x = 40	N/A	Asn1333, Cys273, Asn700
				y = 14.360	size_y = 40		Ser699, Ser722, Asp745
				z = −1.182	size_z = 40		Asn720, Asp1661, Ile1637
							Tyr764, Leu743, Asn1635
							Cys789, Glu1328, Ser768
							Arg815, Ile1326, Thr1327
							Ile1325

(*): The most stable bioactive on a target.

**Table 8:** Comparative binding energy between the most stable bioactive(s) and positive control(s) on the PPAR signaling pathway.

Compounds	PubChem ID	Docking Score (kcal/mol)					
		PPARA (PDB ID: 3SP6)	PPARD (PDB ID: 5U3Q)	PPARG (PDB ID: 3E00)	FABP3 (PDB ID: 5HZ9)	FABP4 (PDB ID: 3P6D)	NR1H3 (PDB ID: 2ACL)
**β-Amyrone**	612782	−16.1					
^(1)^ Clofibrate	2796	−6.4					
^(2)^ Gemfibrozil	3463	−6.3					
^(3)^ Ciprofibrate	2763	−5.4					
^(4)^ Bezafibrate	39042	−5.8					
^(5)^ Fenofibrate	3339	−5.4					
**β-Stigmasterol**	6432745		−10.8				
^(6)^ Cardarine	9803963		−8.5				
**β-Amyrone**	612782			−14.0			
^(7)^ Pioglitazone	4829			−7.7			
^(8)^ Rosiglitazone	77999			−7.4			
^(9)^ Lobeglitazone	9826451			−7.3			
**β-Amyrone**	612782				−21.5		
**β-Amyrone**	612782					−13.2	
**β-Amyrone**	612782						−15.4
^(10)^ GW3965	447905						−11.9
^(11)^ T0901317	447912						−8.2

(1)–(5): PPARA agonists, (6): PPARD agonist, (7)–(9): PPARG agonists, (10)–(11): NR1H3 agonists.

**Table 9:** Comparative binding energy between the most stable bioactive(s) and positive control(s) on the PI3K-Akt signaling pathway.

Compounds	PubChem ID	Docking Score (kcal/mol)						
		AKT1 (PDB ID: 3O96)	IL6 (PDB ID: 4NI9)	VEGFA (PDB ID: 3V2A)	PRKCA (PDB ID: 3IW4)	FGF1 (PDB ID: 3OJ2)	FGF2 (PDB ID: 1IIL)	PHLPP1 (N/A in the PDB)
**Neotocopherol**	86052	−7.5						
^(12)^ AT13148	24905401	−6.9						
^(13)^ Afuresertib	46843057	−6.9						
^(14)^ Alliin	87310	−4.8						
**Xanthosine**	64959		−7.4					
^(15)^ APX-115 free base	51036475		−7.2					
^(16)^ Resatorvid	11703255		−7.1					
^(17)^ Myrislignan	21636106		−7.1					
^(18)^ Muscone	10947		−6.7					
^(19)^ 2′,5′-Dihydroxyacetophenone	10279		−6.5					
^(20)^ α-Cyperone	6452086		−6.3					
^(21)^ Veratric acid	7121		−6.1					
^(22)^ Triolein	5497163		−5.5					
^(23)^ Methylthiouracil	667493		−5.4					
^(24)^ Falcarindiol	5281148		−5.2					
**Ethyl palmitate**	12366			−6.4				
^(25)^ BAW2881	16004702			−7.6				
**Ethyl palmitate**	12366				−6.4			
^(26)^ Midostaurin	9829523				−11.0			
**Sitostenone**	5484202					−8.5		
^(27)^ Suramin	5361					−15.4		
**β-Amyrone**	612782						−14.4	
^(28)^ PD 166866	5328127						−8.3	
**Neotocopherol**	86052							−7.2

(12)–(14): AKT1 antagonists, (15)–(24): IL6 antagonists, (25): VEGFA antagonist, (26) PRKCA antagonist, (27) FGF1: antagonist, (28) FGF2 antagonist.

## Data Availability

All data generated or analyzed during this study are included in this published article (and its Supplementary Materials).

## Supplementary Materials

The following are available online at https://www.mdpi.com/article/10.3390/cimb43030133/s1, Table S1: The 154, 466, and 154 targets from SEA, STP, and overlapping targets between SEA and STP, respectively. Table S2: The number of 3028 targets associated with obesity and the number of 85 final targets.

## Abbreviations

cAMP	cyclic Adenosine MonoPhosphate;
CS	Corn Silk;
DLCs	Drug Like Compounds (DLCs);
DM	Diabetes Mellitus;
FGF1	Fibroblast Growth Factor 1;
FGF2	Fibroblast Growth Factor 2;
GC-MS	Gas Chromatography Mass Spectrometry;
GLP-1	Glucagon-Like Peptide-1;
HIF-1	Hypoxia Inducible Factor-1;
HFWD	High-Fat Western-style fat Diet;
MAPK	Mitogen-Activated Protein Kinase;
MDT	Molecular Docking Test;
PI3K-Akt	Phosphoinositide 3-Kinase–Protein Kinase B;
PPAR	Peroxisome Proliferator-Activated Receptor;
PPARA	Peroxisome Proliferator-Activated Receptor Alpha;
PPARD	Peroxisome Proliferator-Activated Receptor Delta;
PPARG	Peroxisome Proliferator-Activated Receptor Gamma;
PPI	Protein Protein Interaction (PPI);
Rap1	Repressor activator protein 1;
RAS	Renin Angiotensin System;
SEA	Similarity Ensemble Approach;
STB	Signaling pathways-Targets-Bioactives;
STP	SwissTargetPrediction;
TPSA	Topological Polar Surface Area;
VEGFA	Vascular Endothelial Growth Factor A.
